# Dopaminergic versus anticholinergic treatment effects on physiologic complexity of hand tremor in Parkinson's disease: A randomized crossover study

**DOI:** 10.1111/cns.14516

**Published:** 2023-10-31

**Authors:** Yusha Cui, Dongning Su, Junjiao Zhang, Joyce S. T. Lam, Shuangshuang Cao, Yaqin Yang, Yingshan Piao, Zhan Wang, Junhong Zhou, Hua Pan, Tao Feng

**Affiliations:** ^1^ Department of Neurology Beijing Tiantan Hospital, Capital Medical University Beijing China; ^2^ China National Clinical Research Center for Neurological Diseases Beijing China; ^3^ Pacific Parkinson's Research Centre, Djavad Mowafaghian Centre for Brain Health University of British Columbia Vancouver British Columbia Canada; ^4^ Hinda and Arthur Marcus Institute for Aging Research Hebrew SeniorLife Roslindale Massachusetts USA; ^5^ Harvard Medical School Boston Massachusetts USA

**Keywords:** anticholinergic, cognitive load, levodopa, multiscale entropy, Parkinsonian tremor

## Abstract

**Aims:**

Parkinsonian tremor (PT) is regulated by numerous neurophysiological components across multiple temporospatial scales. The dynamics of tremor fluctuation are thus highly complex. This study aimed to explore the effects of different medications on tremor complexity, and how the underlying factors contribute to such tremor complexity.

**Methods:**

In this study, 66 participants received a 2‐mg dose of benzhexol or a pre‐determined dose of levodopa at two study visits in a randomized order. Before and after taking the medications, tremor fluctuation was recorded using surface electromyography electrodes and accelerometers in resting, posture, and weighting conditions with and without a concurrent cognitive task. Tremor complexity was quantified using multiscale entropy.

**Results:**

Tremor complexity in resting (*p* = 0.002) and postural condition (*p* < 0.0001) was lower when participants were performing a cognitive task compared to a task‐free condition. After taking levodopa and benzhexol, participants had increased (*p* = 0.02–0.03) and decreased (*p* = 0.03) tremor complexity compared to pre‐medication state, respectively. Tremor complexity and its changes as induced by medications were significantly correlated with clinical ratings and their changes (*β* = −0.23 to −0.39; *p* = 0.002–0.04), respectively.

**Conclusion:**

Tremor complexity may be a promising marker to capture the pathophysiology underlying the development of PT, aiding the characterization of the effects medications have on PT regulation.

## Graphical abstract

As quantified by multiple scale entropy (MSE), tremor complexity may be a promising marker to capture the pathophysiology underlying the development of Parkinsonian tremor (PT), and help characterize the effects of medication on the complex regulation of PT.

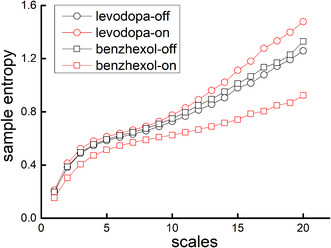

## 1 INTRODUCTION

Parkinson's disease (PD) is a common age‐related neurodegenerative disease.^1^ Parkinsonian tremor (PT) is one of the most significant motor symptoms of PD and oftentimes manifests in hands, severely diminishing the functional independence of those affected by PD. The pathogenesis of PT is highly complex and is attributed a number of changes at both molecular and systems levels over time, such as the build‐up of a‐synuclein,^2^ mitochondrial dysfunction,^3^ loss of dopaminergic neurons,^4^ dysfunction of the nigrostriatal system,^3^ emotional stress,^5^ and diminished cognitive function.^6^

Different types of medications, including levodopa and anticholinergics, have demonstrated great therapeutic potential for PT. However, inconsistent observations of the therapeutic effects of these medications have been reported. For example, Milanov et al. reported that levodopa was more pronounced in reducing the amplitude of PT than anticholinergics,^6^ while Sahoo et al. found that anticholinergics induced greater improvement in Unified Parkinson's Disease Rating Scale‐III (UPDRS‐III) tremor scores (i.e., lower PT severity) than levodopa.^7^ Moreover, the medication effects oftentimes interfere with underlying factors contributing to the development of PT, such as cognitive load. For instance, Zach et al. observed that cognitive load reduced the effectiveness of levodopa medication on alleviating PT.^8^, ^9^ Thus, there is a need to more explicitly characterize the complex regulatory procedure of PT, in order to better understand how different medications, of which the therapeutic mechanisms are distinct (e.g., levodopa supplements the depleted dopamine caused by the loss of dopaminergic neurons,^10^ while anticholinergics block the binding of acetylcholine to M4 muscarinic receptors^11^), influence PT.

Hand tremor, a rhythmic and involuntary oscillation of the hand, is dependent upon numerous underlying elements interacting with a vast array of inputs across multiple scales of time and space.^12^ The dynamics of hand tremor are therefore non‐random (e.g., like Gaussian white noise), but “complex,” consisting of physiologically meaningful patterns in the tremor fluctuations that reflect the adaptive capacity to external stressors.^13^ The presence of PD oftentimes disrupts this complex multiscale regulation, leading to diminished adaptive capacity of the regulatory procedure of tremor to “stressors” (e.g., additional cognitive load). Recent research efforts have implemented techniques from nonlinear dynamics theory to quantify the complexity of tremor fluctuation (i.e., tremor complexity),^14^ and linked this characteristic to important health‐related conditions such as aging and disease states.^15^ In our previous study, for example, we quantified the hand tremor complexity using multiscale entropy (MSE)^16^ and observed that tremor complexity can help differentiate between individuals with PT and those with essential tremor (ET),^16^ while traditional tremor measures (e.g., dominant frequency, amplitude) based upon single‐scale measurements could not. Moreover, we observed that lower tremor complexity was closely associated with greater disease severity as assessed by UPDRS‐III.^16^ Taken together, these findings suggest that tremor complexity may be a promising marker that can capture the changes of tremor regulation pertaining to disease development. Still, how a “stressor” (e.g., cognitive load) influences tremor complexity in PT is not clear, and the effects of anti‐PT medications on tremor complexity have not been explicitly examined and compared.

Therefore, in this randomized controlled and within‐subject crossover study, we characterized tremor complexity in different conditions (e.g., resting, posture, and performing cognitive tasks) in a group of participants with clinically diagnosed PT, and subsequently examined the effects of levodopa and anticholinergic medications on tremor complexity. We hypothesized that: (1) compared to a task‐free condition, tremor complexity would be significantly lower when there is a concurrent cognitive task; (2) the effects of two anti‐PT medications on tremor complexity would be significantly different due to their distinct therapeutic mechanisms; and (3) the changes in tremor complexity as induced by medications would be associated with changes in clinical ratings.

## 2 METHOD

### 2.1 Participants

The recruitment of participants was carried out between August 2021 and March 2023 in the Department of Movement Disorders at Beijing Tiantan Hospital, China. This study has been registered in advance on the Chinese Clinical Trial Registry (registration number: ChiCTR2100050984). The study protocol was approved by the Medical Ethical Review Committee of Beijing Tiantan Hospital according to the Declaration of Helsinki.

The inclusion criteria were: (1) clinical diagnosis of PD according to the Movement Disorder Society Clinical Diagnostic Criteria^17^ by at least three experienced neurologists independently and (2) PD patients with a resting tremor score of ≥1 point in at least one arm on Item 17 of the UPDRS‐III. Those who were with any of the following criteria were excluded: (1) neurologic comorbidities (e.g., history of head trauma, hydrocephalus, brain surgery, or brain tumor); (2) psychogenic tremor; (3) known allergy against levodopa‐benserazide or benzhexol; (4) contraindications of taking benzhexol (e.g., glaucoma, prostatic hyperplasia, and urinary retention); (5) cognitive impairment as assessed by the score of Mini‐Mental State Examination (MMSE) <24 or by the points of Frontal Assessment Battery <12; (6) history of claustrophobia; and (7) metal implants that may interfere with the efficacy of medication (e.g., deep brain stimulation or pacemakers).

### 2.2 Design

**FIGURE 1:**
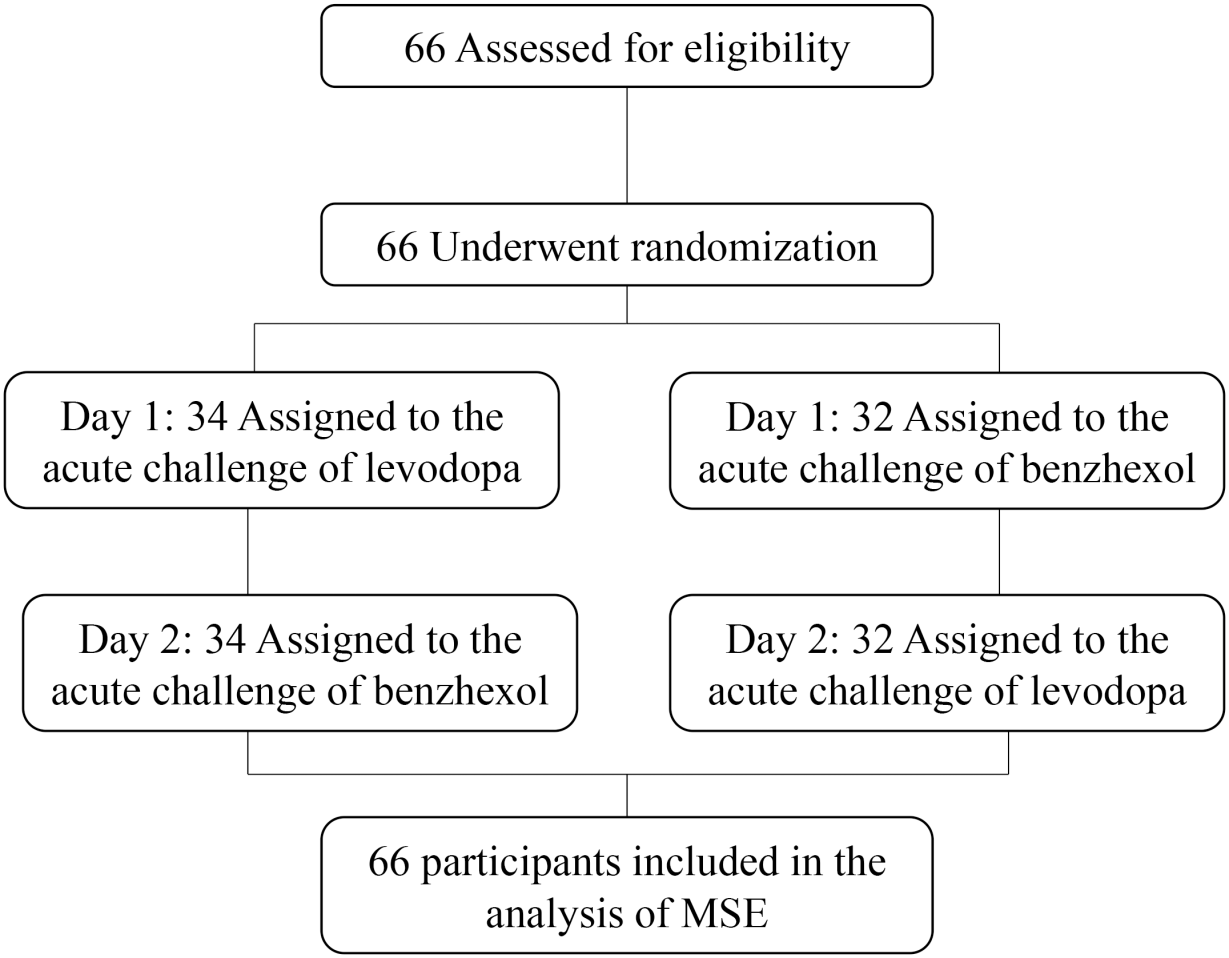
Study protocol.

Each participant completed two study visits consisting of “medication challenge” assessments on two consecutive days. Participants were randomized into one of two parallel trial groups with two sequences of medication administration. In one group, the acute challenge of levodopa was performed on the first visit and that of benzhexol on the second visit; and in the other group, such order of medication administration was reversed (Figure 1). To eliminate the potential effects of the medication used in daily routine, we withdrew participants from their dopaminergic medication(s) at least 12 h and anticholinergic medication(s) at least 24 h before and during the study.

The daily levodopa equivalent dose (LED) was recorded using commonly accepted conversion factors.^18^ At each visit, the medication challenge was performed in the morning after overnight fasting and withholding any anti‐Parkinsonian drugs (i.e., “medication‐off” state). During medication challenge test, participants were instructed to take 150% of their regular morning LED through 200/50 mg dispersible levodopa‐benserazide (i.e., levodopa challenge test),^19^ or take 2 mg of benzhexol (i.e., benzhexol challenge test). The “medication‐on” state was then defined as the time when the maximum beneficial effects of the medication were experienced by the participant and confirmed by research staff via verbal reporting and physical examination in accordance with an established protocol (usually approximately 60 min after medication was taken).^20^ During both the “medication‐on” and “medication‐off” state of each visit, participants underwent a series of clinical assessments and the electrophysiologic tremor assessment.

### 2.3 Clinical tremor assessment

Clinical assessments of tremor consisted of the UPDRS‐III and Fahn‐Tolosa‐Marin Tremor Rating Scale (TRS). The sub‐scores of Items 15–18 in UPDRS‐III were used to assess the specific medication response of tremor. The percent changes in these clinical scores from “medication‐off” to “medication‐on” were also calculated and used in the following analyses.

### 2.4 Electrophysiologic tremor assessment

During the tremor assessment, a six‐channel Nicolet EDX system (Nicolet company, US) with four pairs of surface electromyography (EMG) electrodes and two accelerometers was used to record the tremor fluctuations of each hand (for details, see Appendix S1). Hand tremor was recorded in three trials of 30 s within each of the following six conditions: (1) the forearms rested on two pillows while hands and fingers were unsupported (i.e., REST); (2) the wrist and fingers were straightened to a horizontal plane (i.e., POSH); (3) the arms were lifted and stretched to shoulder level in the horizontal plane with extension of the wrists and fingers (i.e., POSTURE); (4) the upper extremities were in the REST condition (i.e., (1)) while participant were asked to perform a cognitive task (i.e., COG); (5) the upper extremities were in the “POSH” condition while the participants were performing a cognitive task (i.e., DUAL‐TASK); and (6) the upper extremities were in the POSTURE condition (i.e., (3)) while the participants were holding a sandbag weighted 1000 g (WEIGHT). The cognitive task we employed here involved counting backward by seven from a random three‐digit number. Research staff provided a random number to the participants at the beginning of the trial and asked them to count aloud continuously. If any errors were made by the participants, they were instructed to continue counting from that number without stopping.

### 2.5 Multiscale entropy (MSE)

The continuous accelerometer and EMG data were recorded and used in the following analysis. After data preprocessing (for details, see Appendix S1),^16^, ^21^ we used MSE to quantify the tremor complexity. MSE quantifies the physiological complexity of the fluctuations by calculating the sample entropy of the coarse‐grained time series over multiple scales. To capture the multi‐scale dynamics of the tremor, the pre‐processed tremor time series were “coarse‐grained” on different scales of time. Specifically, the time series were divided into nonoverlapping windows of length equaling a scale factor, τ, here ranging from 1 to 30 data points^22^, ^23^ (i.e., Scale 1–30). Sample entropy was then calculated on each of the coarse‐grained series by obtaining the negative natural logarithm of the conditional probability of a time series that repeated itself within a tolerance r for m points (pattern length) as well as of a time series that repeated itself for m + 1 points without self‐matches. In accordance with previous studies, the sample entropy of each coarse‐grained time series was computed by choosing m = 2 and r = 15% in this study.^22^, ^23^ Tremor complexity was then obtained by calculating the area under the MSE curve. Lower MSE reflected lower tremor complexity. The averaged tremor complexity of left and right hand was then used in the following analyses.

The dominant frequency of tremor fluctuation, a traditional metric of tremor, was also obtained and used in subsequent analyses.

### 2.6 Statistical analysis

Analyses were performed using JMP 16 software (SAS Institute, Cary, NC). Data were presented using mean and standard deviation (SD) for continuous variables. The significance level was set at *p* < 0.05. The normality of the data distribution was examined using the Kolmogorov–Smirnov test.

To examine if there were potential carry‐over effects on the clinical characteristics (e.g., total score of UPDRS‐III) and tremor complexity, we used one‐way analysis of variance models when the data were normally distributed. The model factor was visit (i.e., Visit 1 or 2). When the data did not follow a normal distribution or showed unequal variances, we used the Kruskal–Wallis test.

To examine the effects of a cognitive task on tremor complexity, we used separate one‐way analysis of covariance (ANCOVA) models when the data were normally distributed. The model factor was condition, which was, REST versus COG and POSH versus DUAL‐TASK, respectively. Age, sex, disease duration (i.e., number of years since disease was first clinically diagnosed as obtained from medical records), disease severity as assessed by the total score of UPDRS‐III, and cognitive function as assessed using MMSE score were included as covariates. Similar models were used to examine the effects of cognitive task on dominant frequency of tremor. When the data did not follow a normal distribution or showed unequal variances, we used the Kruskal–Wallis test.

To examine the effects of the two anti‐PT medications on tremor complexity, we used one‐way ANCOVA models when the data were normally distributed. The mode factor was medication state (i.e., off and on) and the dependent variable was tremor complexity. Separate models were used to examine the effects of levodopa and benzhexol. Age, sex, disease duration, the total score of UPDRS‐III, and the task condition (i.e., REST, COG, POSH, DUAL‐TASK, POSTURE, and WEIGHT) were included as covariates in these models. Secondarily, we used similar models to examine the effects of medication on tremor complexity in each task condition. Additionally, similar models were used to examine the effects of medications on the total scores of UDPRS‐III and TRS, and dominant frequency of tremor. When the data did not follow a normal distribution or showed unequal variances, we used the Kruskal–Wallis test.

To compare the effects between two medications on tremor complexity, we calculated the percent change in tremor complexity from the “medication‐off” to “medication‐on” state in each task condition and used separate one‐way ANCOVA models to compare the effects of the two medications. The model factor was medication (i.e., levodopa and benzhexol). Age, sex, disease duration, and the total score of UPDRS‐III were included as covariates in these models. When the data did not follow a normal distribution or showed unequal variances, we used the Kruskal–Wallis test.

To examine the association between tremor complexity in “medication‐off” state and clinical characteristics (i.e., total score of UDPRS‐III and score of items related to tremor in UPDRS‐III, and TRS score), separate linear regression models were used. Age, sex, visits (i.e., Visit 1 and Visit 2), disease duration, and task condition were included as the covariates in these models.

To examine the association between the percent changes in tremor complexity as induced by medications and those of clinical characteristics (i.e., percent changes in UPDRS‐III total and sub‐score of tremor, and TRS scores), separate linear regression models were used in the task conditions under which medications induced significance changes in tremor complexity. Age and disease duration were included as covariates.

## 3 RESULTS

All 66 participants successfully completed the assessments. No side effects or adverse events were reported (for details of demographic or clinical information, see Appendix S1).

### 3.1 The effects of cognitive task on tremor complexity

The ANCOVA models demonstrated that in the “medication‐off” state, compared to REST and POSH, the tremor complexity in COG (*p* = 0.002) and DUAL‐TASK (*p* < 0.0001) was significantly lower, respectively. This suggested that the additional cognitive task altered the regulation of tremor fluctuation, resulting in lower tremor complexity. These observations were independent from age, sex, disease duration, the total score of UPDRS‐III, and MMSE score. Similar models showed that the dominant frequency significantly increased in COG compared to REST condition (*p* = 0.001), but did not significantly change between POSH and DUAL‐TASK (*p* = 0.15).

### 3.2 The effects of different medications on tremor complexity and other clinical characteristics

**FIGURE 2:**
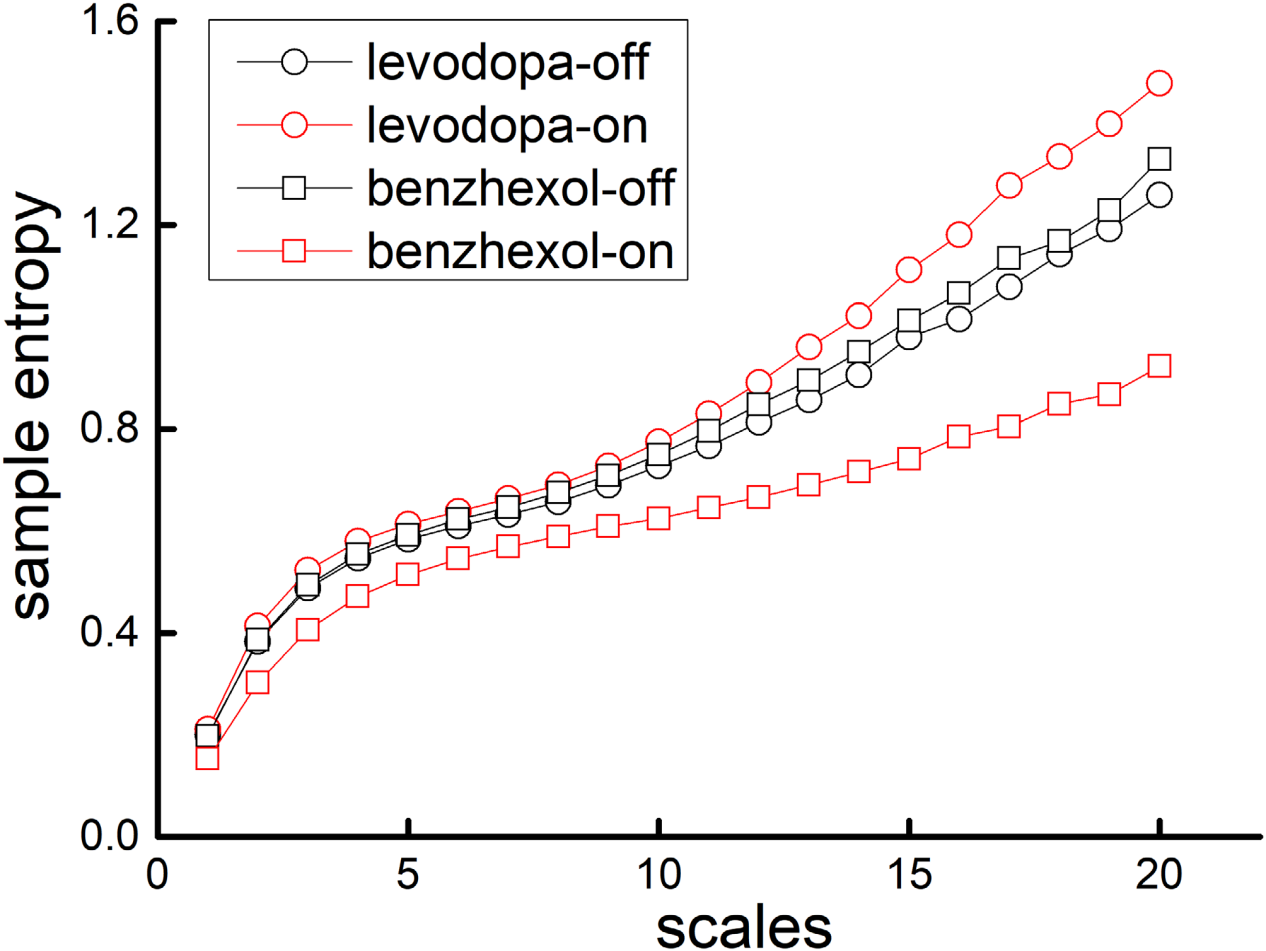
The MSE curves in “medication‐on” (in red) and “medication‐off” (in black) states under levodopa or benzhexol from one representative participant. The entropy was similar at Scale 1. With the increase of the scale, the entropy changed in different directions. Compared to “medication‐off” state, the entropy after taking levodopa increased, while after taking benzhexol, the entropy decreased. This suggests distinct effects of these two medications on tremor complexity.

Figure 2 shows the MSE curves in “medication‐on” and “medication‐off” states under levodopa or benzhexol from one representative participant. The entropies were similar between medication states in small scales (e.g., Scale 1). With the increase of scale, it shows that the entropies changed in different directions, that is, compared to “medication‐off” state, after taking levodopa, the entropy increased; however, after taking benzhexol, the entropy decreased, indicating different effects of these two medications on tremor complexity.

The ANCOVA models demonstrated that at the levodopa visit, compared to “medication‐off” state, the tremor complexity was significantly increased in “medication‐on” state (*p* = 0.007); however, at the benzhexol visit, compared to “medication‐off” state, there was a decreasing trend of tremor complexity toward significance in “medication‐on” state (*p* = 0.07). These observations were independent from age, sex, disease duration, the total score of UPDRS‐III, and the task condition. Furthermore, the models within each task condition showed that levodopa induced a significant increase of tremor complexity in REST (*p* = 0.02), POSH (*p* = 0.02), WEIGHT (*p* = 0.02), COG (*p* = 0.03), DUAL‐TASK (*p* = 0.02), and POST (*p* = 0.03) conditions, whereas benzhexol induced a significant decrease of tremor complexity in POSH (*p* = 0.03), and DUAL‐TASK (*p* = 0.03), but not in any other conditions (*p* = 0.24–0.51). Similar models showed that both medications improved disease severity as assessed by significantly lower total scores of UPDRS‐III (levodopa: *p* < 0.0001; benzhexol: *p* = 0.01), UPDRS‐III tremor score (levodopa: *p* < 0.0001; benzhexol: *p* = 0.0005), and TRS (levodopa: *p* < 0.0001; benzhexol: *p* = 0.008); however, both medications did not induce significant changes in the dominant frequency of tremor (*p* = 0.10–0.87) (Table 1).

**TABLE 1:** Tremor complexity and frequency in “medication‐on” and “medication‐off” states.

	Benzhexol			Levodopa		
	OFF	ON	*p* value	OFF	ON	*p* value
Age (years)	64.3 ± 8.3					
Sex (*n* = female, %)	54%					
MMSE score	27.5 ± 4.5					
Total score of UPDRS‐III	36.5 ± 15.6	31.4 ± 15.5	0.006**	36.9 ± 14.9	24.6 ± 11.3	<0.001***
UPDRS‐III tremor score	9.1 ± 4.5	6.7 ± 4.2	<0.001***	9.3 ± 4.5	4.9 ± 3.7	<0.001***
TRS	17.8 ± 11.5	13.9 ± 11.0	0.002**	19.4 ± 13.2	11.1 ± 9.4	<0.001***
Tremor complexity						
REST	8.5 ± 1.6	8.7 ± 1.7	0.45	8.5 ± 1.6	9.0 ± 1.9	0.02*
COG	7.7 ± 1.4	7.9 ± 1.2	0.51	7.9 ± 1.6	8.3 ± 1.8	0.02*
POSH	9.4 ± 1.6	9.0 ± 1.4	0.03*	9.2 ± 1.4	9.7 ± 1.4	0.02*
DUAL‐TASK	8.5 ± 1.6	8.4 ± 1.3	0.03*	8.5 ± 1.5	8.6 ± 1.5	0.03*
POSTURE	9.3 ± 2.0	9.3 ± 1.3	0.24	9.2 ± 1.5	9.5 ± 1.6	0.02*
WEIGHT	8.5 ± 1.5	8.3 ± 1.3	0.36	8.5 ± 1.7	8.9 ± 1.5	0.03*
Dominant frequency of tremor						
REST	4.7 ± 0.8	4.7 ± 0.7	0.68	4.8 ± 0.7	4.7 ± 0.7	0.25
COG	5.0 ± 0.7	5.0 ± 0.8	0.52	5.2 ± 1.1	4.9 ± 0.6	0.10
POSH	5.9 ± 1.2	6.0 ± 1.3	0.37	5.8 ± 1.3	5.6 ± 1.1	0.65
DUAL‐TASK	5.5 ± 1.1	5.7 ± 1.3	0.79	5.7 ± 1.2	5.6 ± 1.2	0.64
POSTURE	6.3 ± 2.6	6.1 ± 1.4	0.66	6.0 ± 1.4	6.0 ± 1.7	0.49
WEIGHT	6.1 ± 1.6	6.0 ± 1.6	0.87	6.3 ± 1.8	6.3 ± 1.8	0.73

*Note*: Values are represented as the mean ± standard deviation (SD).

Abbreviations: OFF, medication‐off state; ON, medication‐on state.

* *p* < 0.05;

** *p* < 0.01;

*** *p* < 0.001.

Next, we calculated the percent change in tremor complexity from “medication‐off” to “medication‐on” state in each task condition. Generally, levodopa induced an increase while benzhexol induced a decrease in tremor complexity. The one‐way ANCOVA model showed significant differences in the percent changes in tremor complexity in POSH (*p* < 0.0001), DUAL‐TASK (*p* = 0.0009), and WEIGHT (*p* = 0.001), but not in any other conditions (*p* = 0.36–0.41). These observations were independent from age, sex, disease duration, and the total score of UPDRS‐III (Table 2).

**TABLE 2:** Percent changes in tremor complexity from “medication‐off” to “medication‐on” state.

Medication status (%)	Benzhexol	Levodopa	*p*‐value
REST	0.008	4.4	0.36
COG	0.02	4.5	0.37
POSH	−4.4	4.6	<0.0001***
DUAL‐TASK	−2.5	2.9	0.0009***
POSTURE	−0.8	2.6	0.41
WEIGHT	−3.1	0.05	0.001**

** *p* < 0.01;

*** *p* < 0.001.

### 3.3 The relationship between tremor complexity and the clinical characteristics

Within “medication‐off” state, linear regression models showed that the tremor complexity was significantly associated with the total score of UPDRS‐III (*β* = −0.36, *p* < 0.0001), UPDRS‐III tremor score (*β* = −0.23, *p* = 0.006), and TRS score (*β* = −0.29, *p* < 0.0001). Participants with greater total score of UPDRS‐III, UPDRS‐III tremor score, and/or TRS score had lower tremor complexity. These associations were independent from age, sex, and disease duration.

**FIGURE 3:**
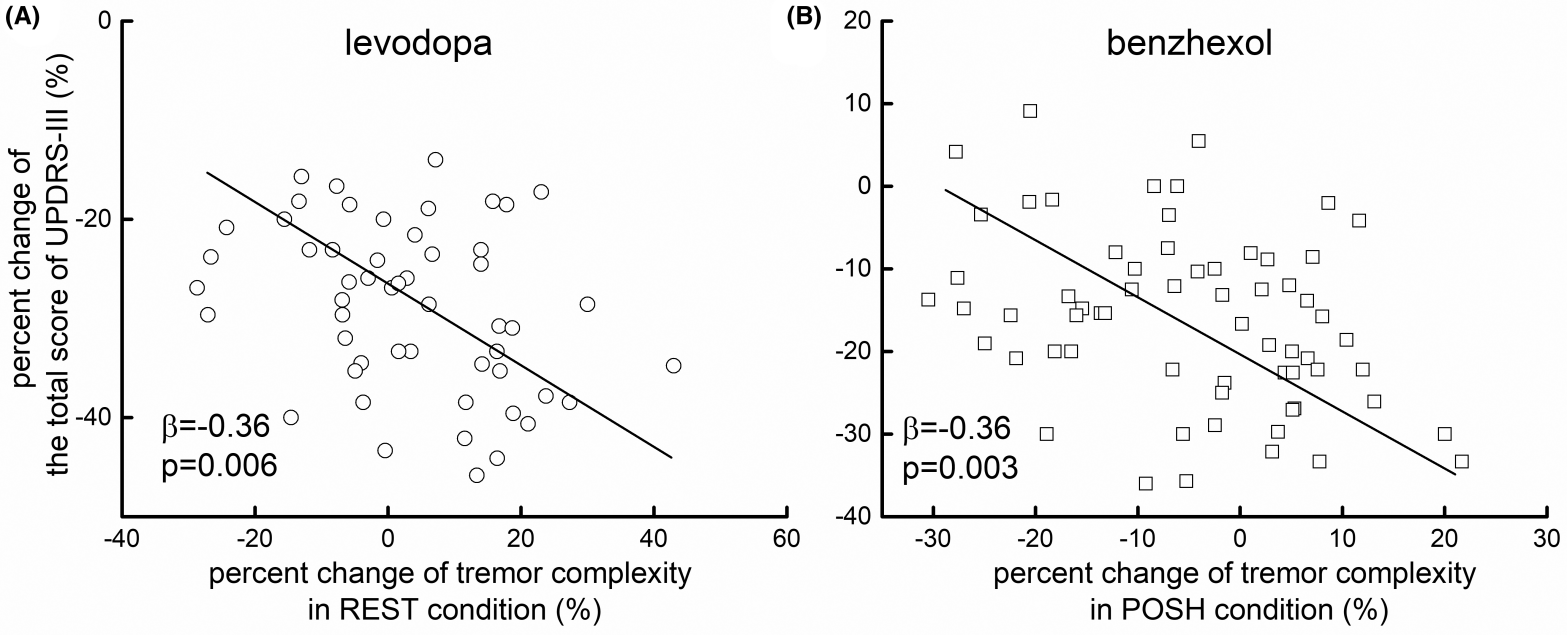
The association between the percent change in tremor complexity in POSH condition as induced by levodopa (A) and benzhexol (B) and that of the total score of UPDRS‐III. Participants with greater increase of tremor complexity after taking levodopa (*β* = −0.25, *p* = 0.04) or benzhexol (*β* = −0.27, *p* = 0.02) had greater reduction of the total score of UPDRS‐III, suggesting greater improvement in the functional performance related to PD.

We then examined the associations between the percent changes in tremor complexity in REST, POSH, DUAL‐TASK, COG, and WEIGHT conditions as induced by levodopa and in POSH and DUAL‐TASK for benzhexol, as in these conditions, the tremor complexity was significantly changed by the respective medication. We also examined associations between the percent changes in the total score of UPDRS‐III, UPDRS‐III tremor score, and TRS score in these conditions. Linear regression models showed that the percent changes in tremor complexity as induced by levodopa in POSH (total score of UPDRS‐III: *β* = −0.25, *p* = 0.04 [Figure 3A]; UPDRS‐III tremor score: *β* = −0.26, *p* = 0.03; TRS score: *β* = −0.31, *p* = 0.01), DUAL‐TASK (total score of UPDRS‐III: *β* = −0.26, *p* = 0.03; UPDRS‐III tremor score: *β* = −0.28, *p* = 0.02; TRS score: *β* = −0.39, *p* = 0.001), WEIGHT (total score of UPDRS‐III: *β* = −0.39, *p* = 0.002; UPDRS‐III tremor score: *β* = −0.31, *p* = 0.01; TRS score: *β* = −0.33, *p* = 0.008) were significantly associated with that of total score of UPDRS‐III, UPDRS‐III tremor score, and TRS score. The percent changes in tremor complexity as induced by benzhexol in POSH condition were also significantly associated with those of the clinical scales (total score of UPDRS‐III: *β* = −0.27, *p* = 0.02 [Figure 3B]; UPDRS‐III tremor score: *β* = −0.28, *p* = 0.02; TRS score: *β* = −0.39, *p* = 0.001). Specifically, participants with greater percent increase of tremor complexity had greater decrease of the scores of these clinical scales, suggesting greater improvement in their clinical performance.

## 4 DISCUSSION

To our knowledge, we here demonstrate for the first time that performing a cognitive task disrupts the multiscale regulation of hand tremor, as reflected by the lower tremor complexity in people suffering from PT. Specifically, two medications induce different changes in tremor complexity—levodopa medication increases while benzhexol decreases tremor complexity. We show that these medication‐induced changes in tremor complexity are closely associated with those of clinical ratings. Together, these observations suggest that tremor complexity may be a promising marker to capture the insights into the pathophysiology underlying PT, aiding the characterization of the effects medications have on PT regulation.

It is observed that performing a cognitive task is associated with lower tremor complexity. Cognitive load has shown to be associated with worsening of PT severity/amplitude.^9^ Two potential pathways of such alteration have been proposed: the “top‐down” mechanism showing that cognitive load amplifies tremor by strengthening the connectivity between the cognitive network and the cerebello‐thalamo‐cortical circuit; and the “bottom‐up” mechanism suggesting that cognitive load is associated with increased arousal, and the ascending arousal system (e.g., brainstem) modulates tremor‐related activity in the ventrolateral nucleus of thalamus, pars ventralis (VLpv).^24^, ^25^ Therefore, the add‐on of cognitive load may alter the regulation of hand tremor, especially in those with PT, resulting in lower tremor complexity as compared to a task‐free condition. It is worthwhile to further explore whether such alteration in the regulation of tremor is unique to PT, or it also exists in other types of movement disorders (e.g., ET). This will ultimately help us better understand the role of cognitive function in the development of different pathological tremors.

We here observed that two commonly prescribed anti‐PT medications—levodopa and benzhexol—have opposite effects on tremor complexity, that is, levodopa increases tremor complexity while benzhexol decreases it. These observations suggest that tremor complexity could be a marker to characterize the underlying neurophysiologic changes in the regulation of hand tremor as induced by medications with distinct mechanisms. The tremor may originate from a network consisting of multiple neural oscillators (i.e., tremor in healthy population), or from one most significant oscillator (i.e., PT). The multiple neural oscillators may therefore induce tremor fluctuations with patterns over multiple temporal scales, which is reflected by higher tremor complexity, while the tremor that is induced by one dominant oscillator may produce fluctuations with one or very limited number of patterns and result in lower tremor complexity.^26^, ^27^ Dopaminergic medications can modulate the network of neural oscillators from the simple, one‐oscillator dominated state of PT to the multiple‐oscillator state by normalizing the functional interaction between thalamus and motor cortical areas^28^ and restoring the connectivity of the basal ganglia motor circuit.^29^ This dopaminergic modulation thus increases the inputs and interactions between the underlying components pertaining to the tremor regulation, leading to increased tremor complexity. Anticholinergics (i.e., benzhexol), on the other hand, alleviate tremor and decrease tremor amplitude by inhibiting the activity of multiple brain regions (e.g., cortex, basal ganglia, thalamus, hippocampus, and cerebellum).^30^ This inhibition of functional regions within the brain may diminish inputs and interactions between components of the oscillatory network pertaining to the regulation of tremor, resulting in decreased tremor complexity. It should be noted that we only used levodopa to perform the acute dopaminergic challenge and only measured the short‐term effects of a single‐dose medication. It is worthwhile in future studies to characterize the longer‐term effects (e.g., 4 h following administration with assessments every 30 min) of multiple doses of medication and explore if other types of dopaminergic drugs (e.g., dopamine agonists) induce changes in tremor complexity similar to those of levodopa.

Findings here demonstrate that levodopa induces changes in tremor complexity in both resting and postural conditions, whereas benzhexol induces changes in postural condition only. These are consistent with previous studies indicating that dopaminergic medication is more effective on resting tremor,^31^ the pathogenesis of which is associated with contralateral reduction in striatal dopamine bindings.^32^, ^33^, ^34^, ^35^ Meanwhile, both the vesicular acetylcholine transporter binding in the paracentral lobule and putamen as well as serotonin depletion in the raphe nucleus play an important role in the regulation of postural tremor,^32^, ^36^, ^37^ and cholinergic medications, such as benzhexol, may particularly be effective for postural tremor.^31^ These observations indicate that caution needs to be taken when determining the appropriate medications for tremor that occurs in different conditions.

Tremor complexity and its changes as induced by medications are strongly correlated with clinical ratings and with changes in the ratings as induced by medications, respectively, while traditional measures of tremor do not show such correlations. These results are consistent with the findings from our previous study.^16^ Numerous studies have suggested that the quantification of physiologic complexity of the output fluctuations from neurophysiological procedures may provide novel insights into the complex regulation of these procedures that cannot be captured by traditional measures based upon a single scale.^12^, ^38^ Our study demonstrates that tremor complexity holds great promise to serve as a marker to characterize the disease severity of PT.

In conclusion, our study shows that tremor complexity can be used to assess the influences of cognitive load on the regulation of PT, and that it is sensitive to the distinct mechanisms of different therapeutics for tremor. Additionally, when more future work is completed, it may be possible to develop a reliable and validated reference based upon tremor complexity to help identify the responsiveness of individuals to anti‐PT medications in clinical practice. Altogether, this work shows that by characterizing the tremor complexity, it may provide critical knowledge of the pathogenesis of PT and guide the development of appropriate treatment for different types of PT.

## AUTHOR CONTRIBUTIONS

Study conception: Yusha Cui, Dongning Su, Junjiao Zhang, and Tao Feng. Data collection: Yusha Cui, Junjiao Zhang, Shuangshuang Cao, Yaqin Yang, Yingshan Piao, Zhan Wang, and Hua Pan. Data analysis: Dongning Su, Junjiao Zhang, Yusha Cui, and Tao Feng. Data interpretation: Yusha Cui, Dongning Su, Junjiao Zhang, and Tao Feng. Drafted manuscript: Yusha Cui. Critically reviewed manuscript: Dongning Su, Junjiao Zhang, Tao Feng, and Joyce S. T. Lam. All authors approved the final version of the manuscript and agree to be accountable for all aspects of the work in ensuring that questions related to the accuracy or integrity of any part of the work are appropriately investigated and resolved. All persons designated as authors qualify for authorship, and all those who qualify for authorship are listed.

## FUNDING INFORMATION

This work was supported by the National Natural Science Foundation of China (No. 82071422) and the Natural Science Foundation of Beijing Municipality (No. 7212031).

## CONFLICT OF INTEREST STATEMENT

No conflict of interest.

## CLINICAL TRIAL REGISTRATION

This study has been registered in advance on the Chinese Clinical Trial Registry (registration number: ChiCTR2100050984).

## PATIENT CONSENT STATEMENT

All participants provided written informed consent in order to participate in this study.

## Supporting information


Appendix S1

## Data Availability

The raw data supporting the conclusions of this article will be made available by the authors, without undue reservation.
