# Supplementary material for: Dopaminergic versus anticholinergic treatment effects on physiologic complexity of hand tremor in Parkinson's disease: A randomized crossover study
Source: CNS Neurosci Ther. 2023 Oct 31;30(4):e14516. doi: 10.1111/cns.14516 (PMC11017432; doi:10.1111/cns.14516)
Supplement: Supplementary file 1 — Appendix S1 [file CNS-30-e14516-s001.docx]

## Methods S1

## Electrophysiologic tremor assessment

Participants were seated comfortably in a chair at resting state. Six electrodes were placed bilaterally on the belly of the extensor digitorum communis and flexor carpi radialis muscles of upper limb, and the two reference electrodes were placed on the corresponding distal tendons. Accelerometers were attached to the dorsum of both hands at two centimeters near the third metacarpophalangeal joint.

### Multiscale entropy (MSE)

Empirical mode decomposition was first used to remove the very low or high frequency trends in the raw time series of tremor. To ensure that a sufficient number of physiologically meaningful patterns occurred within the time series, fluctuations at frequencies lower than 0.1 Hz were removed. Additionally, to avoid potential high-frequency artifacts or noise, those at frequencies higher than 30 Hz were also removed.

## Results S1

## Demographic and clinical information of participants

All 66 participants [age (mean ± SD): 64.3 ± 8.3 years; disease duration: 5.1 ± 4.5 years; MMSE: 27.5 ± 4.5; UPDRS-Ⅲ total score: 36.9 ± 15.0; UPDRS-Ⅲ tremor score: 9.3 ± 4.5; TRS: 19.4 ± 13.2] successfully completed the assessments. After taking benzhexol, there was 21.1% (±22.3%) improvement in the TRS score, 14.3% (±12.6%) improvement in UPDRS-Ⅲ score, and 22.9% (±32.8%) improvement in UPDRS-Ⅲ tremor score; and after taking dopaminergic drugs, there was 42.7% (±23.9%) improvement in TRS score, 31.1% (±16.2%) in MDS-UPDRS-III score, and 46.2% (±33.2%) in UPDRS-III tremor score. No significant difference in the total score of UPDRS-Ⅲ, UPDRS-Ⅲ tremor score, TRS score, and tremor complexity in each task condition in “medication-off” state between the two study visits was observed (p=0.74~0.99), suggesting no carry-over effects of the medication from the prior visit on these characteristics.
